# Do the dual-task “8-foot up and go” tests provide additional predictive value for early detection of cognitive decline in community-dwelling older women?

**DOI:** 10.1007/s40520-022-02193-x

**Published:** 2022-07-15

**Authors:** Jingjing Wang, Jin-Tao Hong, Yun Xiang, Chunhua Zhang

**Affiliations:** 1 School of Exercise and Health, Shanghai University of Sport, Yangpu District, 650 Qing Yuan Huan Rd, Shanghai, 200438 China; 2 Shanghai Research Institute of Sports Science (Shanghai Anti-Doping Agency), Shanghai, 200030 China; 3 School of Physical Education, Hubei Engineering University, Xiaogan, 432000 Hubei China

**Keywords:** Dual-task, 8UG, Mobility, Cognitive function, Older adult

## Abstract

**Background:**

The 8-Foot Up and Go (8UG) test is a widely used mobility assessment. Some dual-task mobility assessments have been developed to help detect cognitive decline.

**Aims:**

This study developed a dual-task version of 8UG test to investigate the dual-task 8UG performance and to evaluate the ability of dual-task 8UG test in detecting cognitive decline.

**Methods:**

A total of 101 eligible community-dwelling women aged 60–74 years were grouped into the mild cognitive impairment group (MCI, *n* = 49) and the non-cognitive impairment group (NCI, *n* = 52). The 8UG tests under single-task (ST), manual dual-task (MT), and cognitive dual-task (CT) conditions were performed respectively. The dual-task cost (DTC) and the correct response rate (CRR) were calculated to quantify the dual-task interference.

**Results:**

Participants spent more time in performing the 8UG test under dual-task conditions. No differences were observed between NCI and MCI groups for 8UG parameters under ST and MT conditions (*p* > 0.05). When executing CT, significant differences were found in the number of correct answers and CRR (*p* < 0.05). CRR showed the strongest ability to predict MCI with a cut-off point of 0.50 (71.2% sensitivity and 61.2% specificity).

**Discussion:**

Both manual and cognitive dual-task were found to interfere with the 8UG performance. CRR with cutoff point of 0.50 could be a potential predictor of MCI in community-dwelling older women.

**Conclusions:**

The CRR of the cognitive dual-task 8UG test could be recommended as a potential predictor for the early detection of MCI in community-dwelling older women.

## Introduction

Cognitive impairment in older adults is a common condition and one of the major public health concerns. Age-related cognitive decline is a gradual aging process [1, 2]. Early detection and early treatment may help to delay the progression of cognitive decline [3, 4].

Mobility is found to be interrelated with cognitive function [5]. On the one hand, cognition plays an important role in normal walking [6] and physical mobility [7]. Impaired cognitive function may lead to an increased falls risk [8, 9]. On the other hand, poor mobility performance may predict cognitive decline [10]. In the aging process, the cortical gait control and cognitive function interacted with each other, and might result in motoric cognitive risk syndrome, which is related to major neurocognitive disorders [11]. The mechanism underlying their associations has not yet been completely explained. Even so, the deficit of cognitive function in attention, executive function, and working memory loss have been demonstrated to contribute to poor performance in postural control, mobility and gait [6, 12, 13].

Prior research has found that to maintain postural stability and mobility, increased recruitment of generic cognitive resources is demanded to compensate for the deficit of age-related neuromotor and sensory degeneration in older adults [14]. However, when two or more tasks are demanded to perform concurrently, a competition for common neural structures and potential trade-off of task prioritization tends to be aggravated [15]. The dual-task testing paradigm, which assesses the individuals’ mobility or balance performance while executing another cognitive or physical task concomitantly, was designed by simulating daily social activity (e.g., walking while talking on the cellphone, thinking about something else, or carrying a cup of coffee). In recent years, the dual-task testing paradigm has been shown to have important value in the early detection of cognitive decline [16], fall risk assessment [17, 18], and understanding of the interactions between cognition and motor control [19]. In addition, dual-task performance might help to explain certain relationships, for example, a mediating effect on the association between fear of falling and activities of daily living [20].

The 8-Foot Up and Go (8UG) test, which is widely applied in functional mobility assessment [21], is developed to assess the agility and dynamic balance of older adults [22]. As a modified version of the Timed Up and Go (TUG) test, the 8UG test involves the same phases as the TUG test. These phases are common functional movements of daily life, including sit to stand, walk, turn around, and turn to sit. To address the limitation of the TUG test, the distance of the 8UG test was shortened from 3 m to 2.44 m (8 feet), for the purpose of increasing the test feasibility in areas with limited space. Additionally, to signal the turn-around, the turning line of TUG was replaced by a cone placed at the turning point in the 8UG test [23]. Previous studies have indicated that compared with single-task TUG, the dual-task TUG test has a stronger ability in detecting individuals at high risk for cognitive decline [24, 25] and falls [26, 27]. Although the 8UG test is a widely used modified version of the TUG test, limited experimental evidence can support or refute whether the dual-task 8UG test has the same predictive ability for identifying older adults at high risk for falls or cognitive decline.

Community-dwelling older women exhibit a faster rate of cognitive decline and mobility decline than men [28, 29]. Comparing with young and middle-aged women, older women showed a more significant decrease in mobility performance under dual task conditions [30]. It is therefore necessary to detect early and intervene timely in these aging changes among this population. Given that physical mobility is correlated with cognitive function in older adults, dual-task versions of 8UG tests were developed with the hypothesis that dual-task 8UG performance is correlated with cognitive function. The purposes of this study were to: (1) assess the dual-task interference on 8UG performance; (2) compare single- and dual-task 8UG performance between participants with and without cognitive impairment; and (3) evaluate the ability of dual-task 8UG tests in detecting cognitive decline.

## Methods

### Participants

A total of 101 eligible community-dwelling older women were enrolled in this study. The inclusion criteria were: (1) community-dwelling women aged 60–74 years; (2) being able to walk independently; (3) having normal vision and hearing; and (4) being willing to and capable of providing informed consent. Participants were excluded from the study if they met the following exclusion criteria: (1) suffering from severe heart, lung, and skeletal muscle system diseases, or neurological diseases that seriously affect balance function (e.g., stroke and Parkinson disease), or mental illness (e.g., depression or take psychotropic drugs), or dementia (moderate and above); (2) using assistive devices for walking; and (3) being illiterate.

### Procedures

The data collection was conducted during a single session. After signing the written informed consent, all participants underwent face-to-face interviews conducted by a trained staff member to collect the demographic and health status information including age, education, medical history (hypertension, diabetes mellitus, cardiovascular disease, hyperlipidemia), and self-reported health status (categorized as “very good”, “good”, “fair”, “poor”, and “very poor”). The Beijing version of Montreal Cognitive Assessment (MoCA-BJ) was then used to evaluate the participants’ cognitive function [31]. Body height (cm) and weight (kg) were measured according to standardized procedures [32].

The 8UG test was carried out under three conditions: 1) single-task condition (ST): 8-foot up and go (8UG) test; 2) manual dual-task condition (MT): 8UG and carrying a cup; and 3) cognitive dual-task condition (CT): 8UG and serial subtraction of 7. Two trials were performed under each condition. The order of trails for each participant was chosen randomly to avoid performance bias [33]. In addition, participants were able to undertake a rest period between each trial to reduce the possible effects of fatigue [34].

### The 8UG test

The 8UG test was carried out according to the method described by Rikli and Jones [22]. A standard folding chair with 43 cm seat height was placed against the wall of a gymnasium, and a cone marker was set exactly 8 feet away from the front edge of the folding chair. Once the start signal “go” was verbally given by the assessor, the participant was instructed to get up from the chair, walk straight to the cone marker, turn around, walk back, and sit down as quickly and as safely as possible. The time taken to complete the test from the word “go” was given to the exact instance the participant sat back down on the chair and was recorded as 8UG_ST_ to the nearest 0.01 s.

In the 8UG-MT test, a standard manual task was added in the 8UG test process. In preparation for the 8UG-MT, a glass was placed on a table (70 cm high) beside the testing chair, and filled with water (300 g) [35–37]. When performing 8UG-MT test, the participants were instructed to complete the 8UG test while carrying a glass of water with their dominant hand [27, 35, 37–39]. In addition to recording the time taken to execute 8UG-MT test (recorded as 8UG_MT_), the weight of water spilled out during the test was calculated.

The 8UG-CT test was a subtraction task added in the 8UG test process. In the 8UG-CT test, participants performed a sequential subtraction of seven from a number randomly selected from 50 to 100 when they executing the 8UG task [33, 40]. The time spent in the 8UG-CT test (recorded as 8UG_CT_) was written down, and the number of correct responses in the serial subtraction task was recorded as well.

In both MT and CT dual-task 8UG tests, no prioritized task was given to simulate a real-life situation [33, 41].

### Classification of cognitive impairment

Individuals’ cognitive functions were evaluated one-by-one respectively by several trained instructors in different rooms. As a widely used Chinese version of MoCA, MoCA-BJ has been shown to be able to detect mild cognitive impairments (MCI) in community-dwelling older adults [31, 42]. A cutoff was set at 26 to distinguish participants with a high risk of MCI (total score < 26) and non-cognitive impairment (NCI, at low risk of MCI, total score ≥ 26) [42].

### Data analysis

With the assumption that 8UG performance might be influenced by executing two tasks concurrently, dual-task cost (DTC) was calculated to quantify the dual-task interference [43]. The DTC is determined according to the differences between single- and dual-task 8UG performances. The formulas of DTC under MT and CT were calculated as follows:

$${\text{DTC}}_{{{\text{MT}}}} \,{ = }\,\frac{{\left( {{\text{8UG}}_{{{\text{MT}}}} - {\text{8UG}}_{{{\text{ST}}}} } \right)}}{{{\text{8UG}}_{{{\text{ST}}}} }}{\text{*100\% }}$$

$${\text{DTC}}_{{{\text{CT}}}} \,{ = }\,\frac{{\left( {{\text{8UG}}_{{{\text{CT}}}} - {\text{8UG}}_{{{\text{ST}}}} } \right)}}{{{\text{8UG}}_{{{\text{ST}}}} }}{\text{*100\% }}$$

A positive value (+) of DTC represents a diminished 8UG performance (added time taken) due to dual-task, while a negative value(-) represents an enhanced performance [44]. A higher absolute value of DTC indicates a higher interference effect [45].

Additionally, in the 8UG-CT test, the response accuracy, which is also described as the correct response rate (CRR) [45–47] was calculated as follows:

$${\text{CRR}}\,{ = }\,\frac{{{\text{Number}}\,{\text{of}}\,{\text{correct}}\,{\text{responds}}}}{{{\text{8UG}}_{{{\text{CT}}}} }}$$

The research data were initially recorded on paper forms. Double data entry was conducted in EpiData 3.1 software (EpiData Association, Odense, Denmark) to make sure the accuracy of the data. Statistical analyses of the current study were performed using SPSS 22.0 software (IBM SPSS Statistics, IBM Corporation, NY, USA).

Variables were expressed as counts (percentage) for categorical data, and as mean (standard deviation, SD) for quantitative data. Chi-square tests were used to compare categorical data between groups. Normal distribution of quantitative data was tested using the Shapiro–Wilk test. A repeated measures analysis of variance was conducted with between-factor as cognitive impairment groups (MCI, NCI), within-factor as tasks (8UG_ST_, 8UG_MT_, and 8UG_CT_), and interactions between task and group. Independent two-sample t-tests were used to compare the difference between groups. Also, receiver operating characteristics (ROC) analysis was performed to determine the ability of the 8UG test parameters in detecting MCI. The optimal cut-off value, area under ROC curve (AUC), sensitivity, and specificity were then reported. The significance level was established as 5%.

## Results

The demographic characteristics and health status of participants are summarized in Table 1. The 101 participants (65.6 ± 3.4 years) were classified into two groups based on their MoCA results. Participants with MoCA total score lower than 26 were categorized into the MCI group (*n* = 49, 65.3 ± 3.3 years), while others were categorized into the NCI group (*n* = 52, 65.9 ± 3.6 years). No significant statistical differences were found in characteristics including age, height, weight, BMI, education years, medical history, and self-reported health status between groups (*p* > 0.05). In general, the cognitive function performances of the MCI group were worse than that of the NCI group (*p* < 0.05), except for the orientation dimension.

**Table 1:** Demographic characteristics and health status of participants

Variable	Total *n* = 101	NCI *n* = 52	MCI *n* = 49	*P* value
Age (years), mean (SD)	65.6 (3.4)	65.9 (3.6)	65.3 (3.3)	0.382
Height (cm), mean (SD)	158.5 (4.5)	158.7 (4.6)	158.3 (4.5)	0.647
Weight (kg), mean (SD)	58.8 (6.7)	59.1 (6.6)	58.5 (6.8)	0.674
BMI (kg/m^2^), mean (SD)	23.4 (2.3)	23.4 (2.2)	23.3 (2.4)	0.838
Education years ≤ 12, *n* (%)	89 (88.1%)	43 (82.7%)	46 (93.9%)	0.083
No. of comorbidities				
0	52 (51.5%)	28 (53.8%)	24 (49.0%)	0.625
1	26 (25.7%)	11 (21.2%)	15 (30.6%)	0.277
≥ 2	23 (22.8%)	13 (25.0%)	10 (20.4%)	0.582
Chronic disease, *n* (%)				
Hypertension (yes)	23 (22.8%)	10 (19.2%)	13 (26.5%)	0.382
Diabetes (yes)	6 (5.9%)	2 (3.8%)	4 (8.2%)	0.620
Cardiovascular disease (yes)	10 (9.9%)	7 (13.5%)	3 (6.1%)	0.368
Hyperlipidemia (yes)	5 (5.0%)	4 (7.7%)	1 (2.0%)	0.396
Self-reported health status, *n* (%)				
Very good	21 (20.8%)	10 (19.2%)	11 (22.4%)	0.474
Good	37 (36.6%)	22 (42.3%)	15 (30.6%)	
Fair	43 (42.6%)	20 (38.5%)	23 (46.9%)	
Total score of MOCA-BJ, mean (SD)	25.2 (3.3)	27.8 (1.4)	22.5 (2.5)	< 0.001^*^
Visuospatial/executive function	3.7 (0.9)	4.1 (0.7)	3.3 (1.0)	< 0.001^*^
Naming	2.8 (0.6)	3.0 (0.2)	2.6 (0.8)	0.001^*^
Attention	5.4 (0.9)	5.8 (0.4)	4.9 (1.0)	< 0.001^*^
Language	1.9 (0.9)	2.3 (0.7)	1.4 (0.8)	< 0.001^*^
Abstraction	1.4 (0.7)	1.6 (0.6)	1.1 (0.6)	< 0.001^*^
Delayed recall	3.3 (1.5)	4.3 (0.9)	2.3 (1.5)	< 0.001^*^
Orientation	5.9 (0.3)	6.0 (0.1)	5.9 (0.4)	0.144

*MOCA-BJ* the Beijing Version of Montreal Cognitive Assessment, *SD* standard deviation; NCI refers to participants with non-cognitive impairment; MCI refers to participants with mild cognitive impairment

^*^Significant *p* < 0.05 by Student *t* test or chi-square test compared with NCI

Compared with the 8UG-ST test, participants spent more time in performing the 8UG test under dual-task conditions. Both MT and CT contributed to a significantly prolonged time spending in the 8UG tests compared with ST in community-dwelling older women. The descriptive statistics of 8UG time under three conditions and the results of repeated measures analysis are presented in Table 2. The time of completing the 8UG test under ST ranged from 3.90 s to 6.81 s. The 8UG test under MT and CT led to a significantly increase of time consuming in both the NCI and MCI groups. Statistically significant differences were found between tasks (*p* < 0.001). However, neither statistically significant differences were observed between NCI and MCI group, nor interaction effect between task and group was found (*p* > 0.05).

**Table 2:** Descriptive and repeated measures analyses results of 8UG time under single- and dual-task conditions in Community-Dwelling older women with and without cognitive impairment

Time	NCI		MCI		Task
	Mean (SD)	Range	Mean (SD)	Range	
8UG_ST_(s)	5.35 (0.68)	4.33–6.75	5.34 (0.60)	3.90–6.81	*F* = 486.606
8UG_MT_(s)	7.32 (1.03)	5.53–9.32	7.30 (1.04)	5.48–9.97	*p* < 0.001
8UG_CT_(s)	6.76 (0.94)	5.38–8.75	6.81 (0.92)	5.19–9.26	η_p_^2^ = 0.909

*8UG* 8-Foot Up and Go test, *8UG*_*ST*_ 8UG Time under Single-task, *8UG*_*MT*_ 8UG Time under Manual Dual-task, *8UG*_*CT*_ 8UG Time under Cognitive Dual-task; NCI refers to participants with non-cognitive impairment; MCI refers to participants with mild cognitive impairment

The parameters of the 8UG test performance under ST, MT and CT conditions were displayed in Table 3. In this study, all DTC values were positive (+), which suggested that both MT and CT had a negative impact on 8UG performance. The DTC_MT_ was higher than DTC_CT_ (*p* < 0.05) in both NCI and MCI groups. No statistical differences were found between NCI and MCI groups for 8UG time and DTC value under single- and dual-task conditions. When performing MT, no one spilled water out during the 8UG test. When performing CT, significant differences were observed in the cognitive task performance (the number of correct answers, *p* = 0.001) and cognitive dual-task interference (CRR, *p* = 0.001).

**Table 3:** Comparison of 8UG parameters under single- and dual-task conditions in community-dwelling older women with and without cognitive impairment

Task	Variable	NCI	MCI	*P* value
ST	8UG_ST_ (s)	5.35 (0.68)	5.34 (0.60)	0.936
MT	8UG_MT_ (s)	7.32 (1.03)	7.30 (1.04)	0.937
	DTC_MT_ (%)	37.24 (13.11)	36.89 (12.11)	0.890
	Spilled water (%)	0.00% (0.00%)	0.00% (0.00%)	
CT	8UG_CT_ (s)	6.76 (0.94)	6.81 (0.92)	0.766
	DTC_CT_ (%)	27.23 (16.70)	28.23 (15.37)	0.755
	Number of correct answers (n)	3.77 (0.90)	2.92 (1.41)	0.001^*^
	CRR (n/s)	0.57 (0.16)	0.43 (0.21)	0.001^*^

*8UG* 8-Foot Up and Go test, *ST* Single-task, *MT* Manual Dual-task, *CT* Cognitive Dual-task, *DTC* Dual-task Cost, *CRR* Correct Response Rate; NCI refers to participants with non-cognitive impairment; MCI refers to participants with mild cognitive impairment

^*^Significant *p* < 0.05 compared with NCI

**Fig.1:**
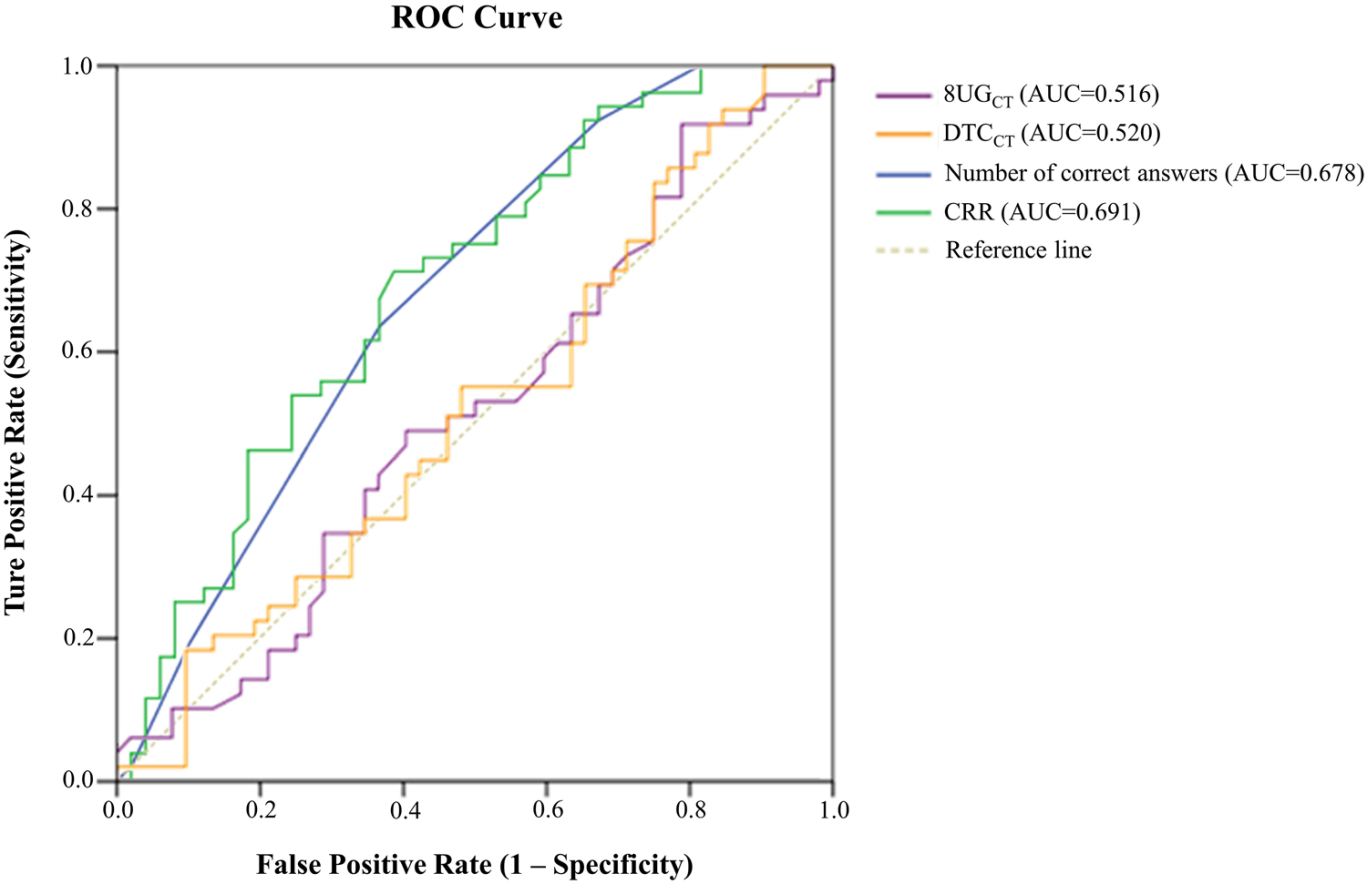
Receiver Operator Characteristic (ROC) Curves to Predict MCI for Parameters of 8UG Test under Cognitive Dule-task (CT) Condition. The predictive ability of 8UG-CT is evaluated by using ROC analysis. Area under the curve (AUC) of 8UG_CT_ (purple curve), DTC_CT_ (yellow curve), the number of correct answers (blue curve) and the correct response rate (CRR, green curve) is 0.516 (*p* = 0.786), 0.520 (*p* = 0.731), 0.678 (*p* = 0.002) and 0.691 (*p* = 0.001). The dotted line represents reference line

On account of that significant differences were detected in the 8UG test performance under CT in the NCI and MCI groups, an ROC analysis of the 8UG test parameters under CT condition was subsequently conducted to compare the ability to identify the participants at high risk of developing MCI. ROC curves for the four 8UG test parameters under CT to predict MCI were shown in Fig. 1. The AUC of 8UG_CT_, DTC_CT,_ number of correct answers and CRR were 0.516, 0.520, 0.678 and 0.691, respectively. Numbers of correct answers and CRR showed good abilities to predict MCI in community-dwelling older women (*p* < 0.05).

Of four parameters, CRR showed the strongest ability to predict MCI. The optimal cut-off values, AUC, sensitivity, and specificity of CRR were showed in Table 4. In order to ensure a high sensitivity (71.2%) and specificity (61.2%) for MCI prediction, the optimal cut-off point of CRR was 0.50.

**Table 4:** Optimal cut-off value, AUC, sensitivity, and specificity of CRR to predict the community-dwelling older women at high risk for developing MCI

CRR	Value
Cut-off point	0.50
AUC	0.691
Sensitivity	71.2%
Specificity	61.2%

*CRR* correct response rate, *AUC* area under the curve

## Discussion

The main findings of this study were: (1) both manual and cognitive dual-task interfered with the performance of the 8UG test, (2) no differences were observed in the 8UG test performance under single- and manual dual-task conditions among participants with and without cognitive impairment, (3) compared with NCI group, participants in MCI group performed worse in certain cognitive dual-task 8UG test parameters, including number of correct answers (cognitive task performance) and CRR (cognitive dual-task interference), and (4) CRR of cognitive dual-task 8UG test could be regarded as a potential predictor of MCI in community-dwelling older women.

In the current study, the dual-task paradigm including manual and cognitive conditions were applied to develop the 8UG test. To our knowledge, this is the first study which aimed to investigate the 8UG performance under dual-task conditions in older women with different levels of cognitive ability. Previously, an animal naming dual-task 8UG test was reported once as one of the cognitive-motor function assessments to measure the motor-cognitive performance in patients with dementia [52]. Considering that the 8UG test is a valid, reliable, and wildly used mobility assessment [48, 49], and mobility in daily life often requires performing multiple tasks simultaneously, there is substantial need for developing dual-task versions of 8UG test to detect early impairment of mobility and cognitive function. With reference to previous studies, two dual-task 8UG tests were developed in this study: manual dual-task (performing the 8UG while holding a cup filled with water concurrently) [35, 38, 50, 51], and cognitive dual-task (performing the 8UG while doing serial subtraction concurrently) [33, 52, 53].

Dual-task might elevate the central resource demand and generate potential resources competition between tasks. It is worth noting that although no task prioritization was given by the instructor, all participants in this study spent longer time in completing the 8UG test under both MT and CT than under ST. The impaired performance of the 8UG test is referred to as dual-task interference [43, 54], and could be quantified by calculating the DTC [43, 55]. In this study, the DTC values under MT and CT were positive (+) attributing to the decrement 8UG performance under dual-task conditions [44]. These findings were consistent with previous findings in dual-task TUG researches [26, 56, 57].

Task interference between motor and posture control tasks occurred often in daily life [41]. For instance, walking while carrying grocery bags or turning back while carrying a cup of coffee. A prior study has reported that participants in different cognitive level groups (NCI, MCI and Alzheimer’s disease) showed different TUG performance under both single- and dual-task conditions [51]. However, in the present study, no group differences between NCI and MCI were found in both single-task 8UG test performance and manual dual-task performance. One possible explanation might be that the participants of this study were younger and healthier, therefore most of them did not yet present severe physical and cognitive function decline.

Numerous studies have confirmed the association between mobility and cognitive function [13, 58]. The age-related mobility decline may be partly compensated by cognitive involvement. Also, the decline of cognitive function may be accelerated due to mobility impairment [58]. Based on this, dual-task performance depends on mobility, cognitive function and the interplay between cognition and motor control [59]. The cognitive dual-task testing paradigm was thus considered as a valuable tool for the early detection of mobility and cognitive decline [17, 60]. In this study, although no between-group statistical differences were found in 8UG_CT_ and DTC_CT_, the MCI group tended to have a longer 8UG time and higher DTC value under CT than ST. In addition, other two parameters of 8UG-CT were observed to have significant between-group differences. These findings revealed that cognitive dual-task led to a decline in mobility performance and the decline varied according to different cognitive abilities.

To further assess the predictive ability of cognitive dual-task parameters, the ROC analysis was conducted. Among four parameters, CRR was the most informative indicator showing an AUC of 0.691 (*p* = 0.001). Although the AUC of CRR was less accurate, the results still implied that CRR was a valuable indicator for early detection of MCI. Previous studies showed that the sensitivity of commonly used instruments for detecting MCI was generally lower. The diagnostic accuracy across studies varied widely with different measurements and cut-points. For instance, the results by Clock Drawing Test and Mini Mental State Examination were inconsistent in detection of MCI [61]. In the current study, the sensitivity of CRR was 71.2% with a cut-off point of 0.50, indicating that CRR was a valuable indicator for early detection of MCI in large population. At the same time, CRR was a derived indicator, which was linked with both mobility and cognitive functions and their interferences. Additionally, considering that significant financial costs and resources consumptions on time, training and diagnosis for detecting MCI [62], it is necessary to conduct research on simple indicators similar to CRR in a larger population.

Studies have suggested that cognitive dual-task testing could be recommended to predict MCI [63, 64] and dementia [65, 66]. Previous studies have found that cognitive function was related to key components of mobility, including gait, turning and transitions [67]. The performance of walking straight-ahead and turning-around was affected by the complexity of secondary tasks in cognitive dual-task TUG [68]. Dual-task TUG has been suggested as an auxiliary diagnostic tool for dementia and MCI [25]. In the 8UG-CT test of this study, significant differences were found in the number of correct answers and CRR between NCI and MCI groups. Based on ROC analysis, CRR, with a cut-off point of 0.50, might be recommended as a potential predictor for the early detection of MCI.

Several limitations should be noted in this study. Firstly, this study is a cross-sectional design. Thus, it is hard to determine a causal relationship. Secondly, the use of MoCA-BJ could also be regarded as a limitation. Among five Chinese versions of MoCA, MoCA-BJ is the most popular version in the mainland China owing to the Mandarin Chinese used in its instruction [69]. Although all participants have adequate ability to understand Mandarin Chinese, language barriers between Mandarin and regional dialects are still encountered during face-to-face interviews sporadically, which might cause misunderstandings. Thirdly, only 101 community-dwelling older women aged 60 to 74 were recruited in this study. It is essential to conduct early detection among men as well, even though women are at higher risk of cognitive and mobility decline than men as we mentioned above. The main findings need to examine both women and men of a wider age bracket with larger sample size.

Future studies are required to examine: the reliability and validity of the dual-task 8UG test, the causality relationship between dual-task 8UG performance, and cognitive function with a prospective longitudinal design, as well as the dual-task 8UG performance in a broader population.

## Conclusions

In conclusion, this study developed dual-task 8UG tests. The findings of the current study showed that both manual and cognitive dual-task interfered with the 8UG performance in both NCI and MCI groups, whereas only cognitive dual-task 8UG test parameters had group differences. Moreover, the CRR of cognitive dual-task 8UG test could be recommended as a potential predictor for the early detection of MCI in community-dwelling older women.

## Data Availability

The datasets generated and analyzed during this study are available from the corresponding author on reasonable request.
